# The women's side of home advantage: comparative analysis in the top seven handball leagues

**DOI:** 10.3389/fspor.2026.1798630

**Published:** 2026-04-24

**Authors:** Moisés Marquina Nieto, Alfonso de la Rubia, Reidel Cordoves Peinado, Raúl Nieto-Acevedo, Guillermo Franco Gimeno, Carlos García-Sánchez

**Affiliations:** 1 Deporte y Entrenamiento Research Group, Facultad de Ciencias de la Actividad Física y del Deporte (INEF), Universidad Politécnica Madrid, Madrid, Spain; 2 Universidad Pedagógica Nacional Francisco Morazán, Universidad Nacional Autónoma de Honduras, Tegucigalpa, Honduras; 3 Facultad de Ciencias Biomédicas y de la Salud, Universidad Alfonso X el Sabio (UAX), Madrid, Spain; 4 Campus de Ciencias de la Vida, Universidad de Nebrija, Madrid, Spain; 5 ETSI de Ingeniería Informática, Universidad Politécnica Madrid, Madrid, Spain

**Keywords:** competitive performance, contextual factors, match location, professional handball, team level

## Abstract

**Introduction:**

This study aimed to: (1) analyse and compare home advantage (HA) and home winning percentage (HW) in the main top European women's handball leagues over the last four seasons; and (2) examine the impact of team level on these indicators to identify patterns of competitive performance.

**Methods:**

A total of 4,744 matches were analysed from the first divisions of France (Starligue), Germany (Bundesliga), Spain (Guerreras Iberdrola), Norway (REMA 1000), Hungary (NB I), Romania (Liga Natională) and Denmark (Kvindeligaen). Each team-season was classified by hierarchical cluster as High-Level Team (HLT), Medium-Level Team (MLT), or Low-Level Team (LLT). HA and HW were calculated and statistically compared between leagues and team levels.

**Results:**

It has been found that HA and HW were observed across all leagues, showing consistent presence but no significant inter-league differences, suggesting that the phenomenon is similarly manifested across European elite women's handball (*F*_6,359_ = 1.55; *p* = 0.162; *η_p_*^2^ = 0.025; small) and HW (*F*_6,359_ = 0.95; *p* = 0.459; *η_p_*^2^ = 0.016; small). Also, showed clear disparities by team level: HLTs outperformed MLTs and LLTs in both indicators (*F*_2,359_ = 11.79; *p* < 0.001; *η_p_*^2^ = 0.062; large) and HW (*F*_2,359_ = 532.35; *p* < 0.001; *η_p_*^2^ = 0.748; large), with all levels differing. Interaction effects were minimal, limited to HA for LLTs, where Germany displayed lower values than Spain and Norway, while intra-league analyses confirmed consistent team-level differences in both HA and HW.

**Conclusion:**

Overall, HA and HW were evident in all seven leagues, suggesting that home advantage is a common phenomenon in elite women's handball, while the competitive level of the opponent exerts a significant influence on performance across leagues.

## 1 Introduction

Performance in European elite women's handball has developed significantly in recent decades, driven by the professionalisation of the leagues and the increase in the level of competition among the continent's top teams (1). In this context, collective performance has become an essential tool for understanding the dynamics of competition and improving the strategic preparation (2). Among the variables studied in team sports are Home Advantage (HA) and Home Win (HW), which directly influence the performance of the teams and their position in the table league (3, 4). The magnitude varies by sport, country and competitive level, but its presence has been documented in almost all team sports, including handball, although to a lesser extent in the women's field (4–7). The HW is a complementary indicator that quantifies the proportion of matches won by a team when playing at home. Although its use is more frequent in descriptive contexts, it has proven useful in observing longitudinal trends and structural differences between leagues (8). As such, incorporating HA and HW allows for a more comprehensive analysis of the dynamics of home games in elite sport settings (9).

HA represents a relational construct that captures the asymmetry between home and away performance relative to total performance or alternatively through goal differentials or win proportions adjusted for total performance. HA addresses the distinct question: “*To what extent is performance at home superior to performance away, relative to overall team performance?*”. In contrast, HW directly answers the question: “*How often do teams win at home?*” and serves as a straightforward, intuitive measure of home success widely employed across team sports research (10, 11). While HW and HA are mathematically related—since both derive from the same match outcomes—they provide complementary but non-identical theoretical information. HW reflects the absolute level of home success, whereas HA emphasizes the relative contribution of home performance to total achievement. Importantly, divergences between HA and HW patterns may arise mechanically from their distinct constructions: for example, teams with strong away performance may exhibit high HW but moderate HA, while teams with poor away performance may show inflated HA despite modest HW. This structural interdependence, rather than substantive behavioural differences, must be considered when interpreting comparative results (12).

Initial studies have identified multiple factors that explain home advantage, suggesting that some factors, such as public support and facility awareness might have similar roles for both sexes (13). Furthermore, the impact of HA and HW in women's leagues may differ from men's leagues due to contextual variables, such as differences in crowd attendance and the competitive structure of leagues or the profile of female players (14).

Research on HA and HW in women's sport has seen significantly less development compared to men's sport. In the case of women's disciplines, the specific literature on HA and HW is more limited. This difference is due to historical, social and economic factors that have influenced the professionalisation and visibility of women's teams, as well as the availability of data and resources for research (15). Evidence suggests that HA and HW values tend to be slightly lower in women's sports than in men's sports (16, 17). In women's team sports such as football (18), basketball (8), and volleyball (19), various studies have confirmed the existence of HA and HW, but with generally lower values compared to their male counterparts (14). This has been attributed mainly to lower attendance by local audiences, less environmental pressure, and a reduced ability of clubs to influence external contextual factors, although in top-level events, professionalisation could level the playing field between the two genders. Furthermore, recent research highlights that HA in women's sports may be more associated with factors such as calendar organisation, group cohesion and club culture than with environmental pressure (20).

In the specific case of handball, both in men's and women's categories, the literature shows that HA and HW are systematically present in the main European leagues, although generally with lower values in women's handball (21, 22). Previous analyses have reported HA values ranging between 54% and 65% in men's leagues (16, 23) and around 52%–58% in women's competitions (24, 25). Likewise, the literature agrees in associating HA and HW with the team level, the competition category, and the overall competitive balance within each league, with a tendency towards a reduction in home advantage as professionalisation and balance between clubs progresses.

Most of the available research has focused on men's competitions, leaving a significant gap with regard to the specific analysis of the main European women's leagues (1). This lack is particularly relevant given the increasing professionalism of women's handball, as well as the consolidation of highly competitive leagues, which bring together many of the best teams and players on the continent (26, 27).

Despite the growing body of research on HA in team sports, most evidence is based on male competitions, with women's contexts receiving limited and fragmented attention. Therefore, the aim of the present study was: (1) to analyse and compare the home advantage (HA) and home winning percentage (HW) in each of the top European women's handball leagues over the last four seasons; (2) to analyse potential differences in HA and HW across leagues and team levels, in order to identify patterns of competitive performance. Therefore, the aim of this study is not to identify causal determinants of home advantage, but to describe how HA and HW co-vary across team levels and elite European women's handball leagues, providing a comprehensive descriptive perspective on home performance patterns. The present study addresses these gaps through four novel contributions: (I) the first multi-league analysis of HA and HW across seven top European women's handball leagues (France, Norway, Hungary, Germany, Spain, Denmark, Romania); (II) four post-COVID seasons (2021–2024) capturing the reintroduction of crowd effects; (III) team-level clustering (HLT, MLT, LLT) to examine competitive hierarchy effects; and (IV) simultaneous analysis of HA and HW to disentangle absolute home success from relative performance asymmetry.

## 2 Materials and methods

### 2.1 Sample

Data were collected from the seven-handball top-leagues based on European Handball Federation (EHF) criteria which combine (1) sporting performance of clubs in European competitions over a defined period, (2) coefficient points assigned by the EHF based on continental results, and (3) national league strength, which considers club consistency and competitive depth (28): Denmark (Kvindeligaen), France (Starligue), Germany (Bundesliga), Hungary (NB I), Norway (Rema 1000), Romania (Liga Natională), and Spain (Guerreras Iberdrola). These leagues consistently appear among the highest-ranked leagues in Europe, representing the most stable professional environments and the strongest clubs in EHF competitions over the past five years. Their inclusion ensures a homogeneous level of elite performance, comparable organisational standards, and reliable longitudinal data, making them the most suitable group for a cross-national analysis of home advantage in top-level women's handball.

All teams were professional squads participating in the top national divisions of each country, composed exclusively of adult players (17 years and above) according to league registration rules. Although the age of majority in Europe is set at 18, the regulations allow underage players to be registered on teams and must be taken into account. Data were extracted from an open-access website (https://www.flashscore.es), widely used for sport results. To collect the data, no permission was required from the entities responsible for the competitions or from the participants because the data is in the public domain and freely accessible. Flashscore was used as a data aggregator, not as the sole source. Match data (dates, venues, results) were verified against official league websites and national federation databases.

**Figure 1:**
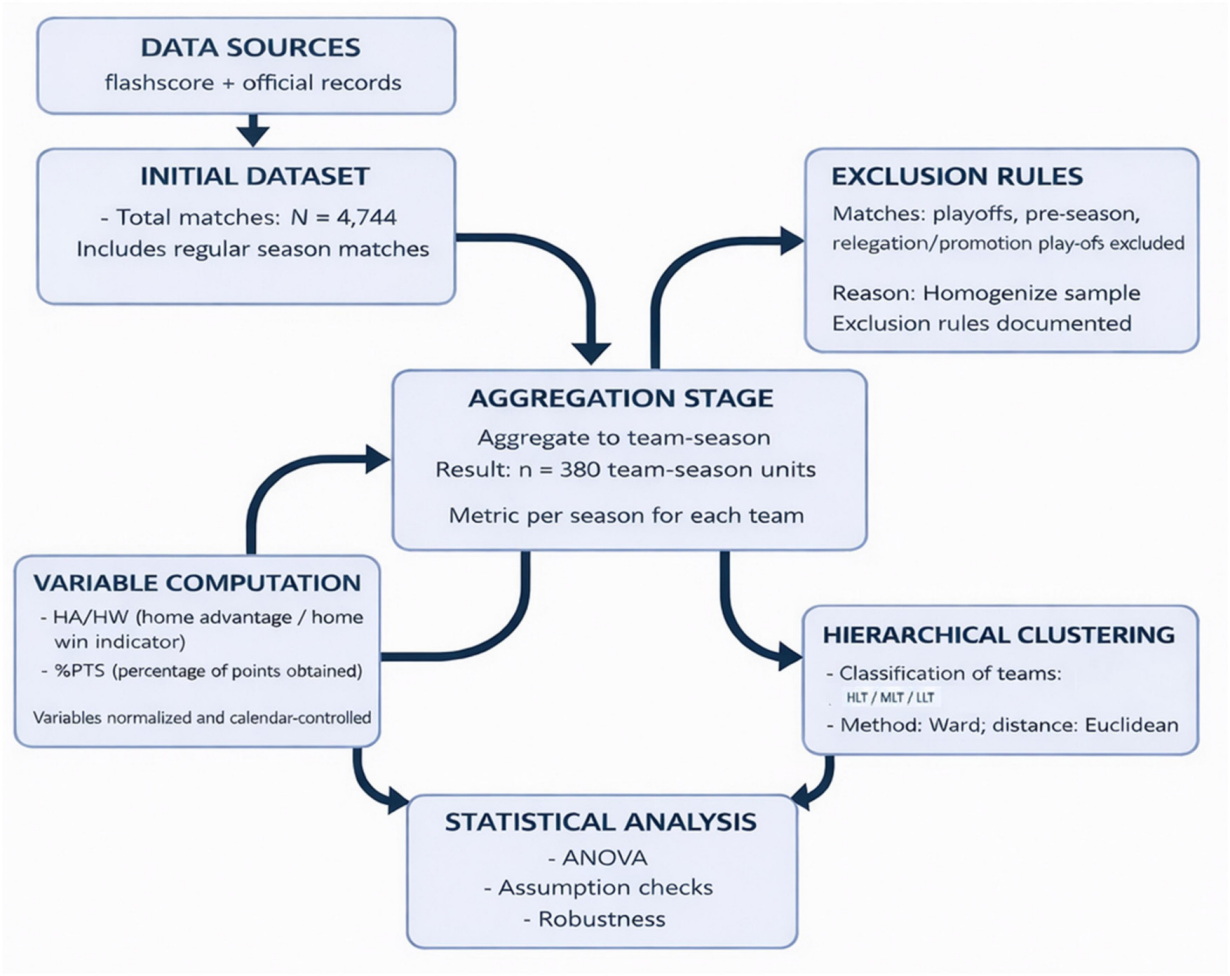
Data processing and analytical workflow.

The data set included four regular seasons (from the 2021–2022 season to the 2024–2025 season) excluding matches played in the preseason, playoffs, and permanence phase. Seasons 2019–2020 and 2020–2021, which were affected by COVID-19 spectator restrictions, were excluded from the analysis. Therefore, all included seasons were played under regular attendance conditions, minimising the potential confounding effect of temporary crowd limitations on home advantage. A total of 4,744 match records were analysed, which were aggregated into 380 team observations per season, constituting the statistical units used in the ANOVA models. Analysing these leagues over the last four seasons (2021/22 to 2024/25) allows to study the recent behaviour of HA and HW under regular competition conditions, without exceptional alterations. Figure 1 shows the flow chart based on the STROBE criteria.

### 2.2 Procedures

Variables collected included season year and competition, as well as the number of home wins/draws/losses, away wins/draws/losses, total wins/draws/losses, total games played at home, total home points, and total points for each team in each season. These data were entered into custom Microsoft Excel spreadsheets (version 24, Microsoft Corporation, Redmond, WA) for further analysis. In these spreadsheets, calculations were performed to determine the HA for each team in each season. Specifically, HA (%) was calculated as [(total home points/total points) × 100], while HW (%) was calculated as [(total home wins/total home games) × 100] (11). Both metrics were selected as complementary indicators that capture distinct but related dimensions of home performance: absolute success rate (HW) vs. relative performance asymmetry (HA). Their structural mathematical relationship is acknowledged, with potential divergences between results interpreted cautiously to account for metric construction effects.

Furthermore, the teams in each competition were ranked according to their performance (i.e., team level) in each season, which was determined by the number of points scored over the course of the season relative to the total possible points that could be scored in each season (e.g., a team scoring 42 points out of a possible 60 points would have a winning percentage of 42/60 = 70%).

Thus, a hierarchical clustering was carried out according to the squared Euclidean distance (29), to stratify the teams according to the ratio between total points obtained and the maximum possible points in each regular season, which is a standard proxy of long-term competitive strength and final ranking. This choice ensured that the team-level classification captured overall performance rather than single-season fluctuations. Hierarchical clustering was conducted using squared Euclidean distance as the similarity measure and Ward's linkage method, which is commonly recommended when the goal is to generate compact and well-separated clusters in continuous performance data. A dendrogram was inspected to determine the optimal number of clusters. The three-cluster solution showed a clear separation in performance ratios and was consistent with the competitive structure of the leagues providing the following results: (1) High-level teams (HLT) (*n* = 78; 20.53% of the total data set; points range = 86.60% ± 8.72%, minimum = 72.22%, maximum = 100%); (2) Medium-level teams (MLT) (*n* = 105; 27.63% of the total data set; points range = 59.92% ± 6.41%, minimum = 50%, maximum = 71.15%); or (3) Low-level teams (LLT) (*n* = 197; 51.84% of total data set; points range = 29.85% ± 12.04%, minimum = 0%, maximum = 48.08%). Team-level clustering based on points percentage was used as a proxy for competitive level; however, this approach does not fully isolate team ability effects.

### 2.3 Statistical analysis

Pearson's correlation was computed to assess the variables relationship between the HA and HW. To determine the interaction of HA and HW as a function of league and team level, a two-factor ANOVA was performed. The two-factor ANOVA was conducted after verifying its underlying assumptions. For normality, the Kolmogorov–Smirnov test (Table 1) was applied to each variable explicitly by the group sizes being >50 in all leagues and complemented by graphical inspection of distributions. The critical assumption of homogeneity of variances was assessed using Levene's test based on mean (Table 2). If this assumption was violated (*p* < 0.05), rendering the ANOVA *F*-ratio potentially incorrect, we implemented appropriate ANOVA corrections, such as non-parametric methods (Kruskal–Wallis) to ensure reliable results. In this context, Kruskal–Wallis statistics were specifically used to test the team-level variable after its assumption (*p* < 0.05). This non-parametric test served as a measure to confirm the data's validity and robustness (30). A posteriori comparison was analysed using the Bonferroni test. If the overall model did not yield significant differences, but with differences in effect size, an exploratory analysis was performed with this test. The lack of a significant interaction effect could be due to the pattern of differences between the groups being relatively constant (i.e., the simple effect was large across multiple levels), which leads to a non-significant *p*-value for the interaction, even though the individual pairwise comparisons do reach the significance threshold. Effect sizes (ES) were also determined for all pairwise comparisons using a partial eta squared (*η_p_*^2^). Interpretation was based on the following criteria (31): *small* = < 0.01; *moderate* = 0.01–0.06; *large* = > 0.06–0.14. For *post hoc* analysis, Cohen's d was calculated and interpreted using Hopkins' categorisation criteria: *d* > 0.2 as *small*, *d* > 0.6 as *moderate d* > 1.2 as *large* and *d* > 2.0 as *very large* (32). The significance level for all statistical tests was set at *p* < 0.05. In addition, standard deviations and confidence intervals are shown for each of the data provided. Descriptive analyses and inferential tests were performed with the SPSS version 30 statistical software package (IBM Corp., Armonk, NY, USA).

**Table 1:** Normality tests based on league and team level.

	Variable	Kolmogorov–Smirnov	df	*p* values		League	Kolmogorov–Smirnov	df	*p* values
HA	Bundesliga (GER)	0.169	54	0.055	HA	HLT	0.108	78	0.054
	Starligue (FRA)	0.145	55	0.076		MLT	0.091	105	0.053
	Guerreras Iberdrola (ESP)	0.156	52	0.067		LLT	0.098	197	0.061
	Kvindeligaen (DEN)	0.184	55	0.052					
	Liga Natională (ROM)	0.217	55	0.051					
	NB I (HUN)	0.129	56	0.102					
	REMA 1000 (NOR)	0.131	53	0.107					
HW	Bundesliga (GER)	0.119	54	0.055	HW	HLT	0.163	78	0.071
	Starligue (FRA)	0.122	55	0.060		MLT	0.133	105	0.056
	Guerreras Iberdrola (ESP)	0.114	52	0.088		LLT	0.102	197	0.053
	Kvindeligaen (DEN)	0.107	55	0.180					
	Liga Natională (ROM)	0.109	55	0.156					
	NB I (HUN)	0.140	56	0.068					
	REMA 1000 (NOR)	0.086	53	0.200					

df, degrees of freedom; HA, home advantage; HW, home win; HLT, high level teams; MLT, medium level teams; LLT, low level teams.

**Table 2:** Test of homogeneity of variance based on league and team level.

	League						Team Level				
	Variable	Levene's test	df1	df2	*p* values		Assumption	Levene's test	df1	df2	*p* values
HA	Based on the mean	1.260	6	373	0.275	HA	Based on the mean	39.406	2	377	0.021
HW	Based on the mean	0.930	6	373	0.474	HW	Based on the mean	12.714	2	377	0.057

df, degrees of freedom; HA, home advantage; HW, home win.

## 3 Results

**Figure 2:**
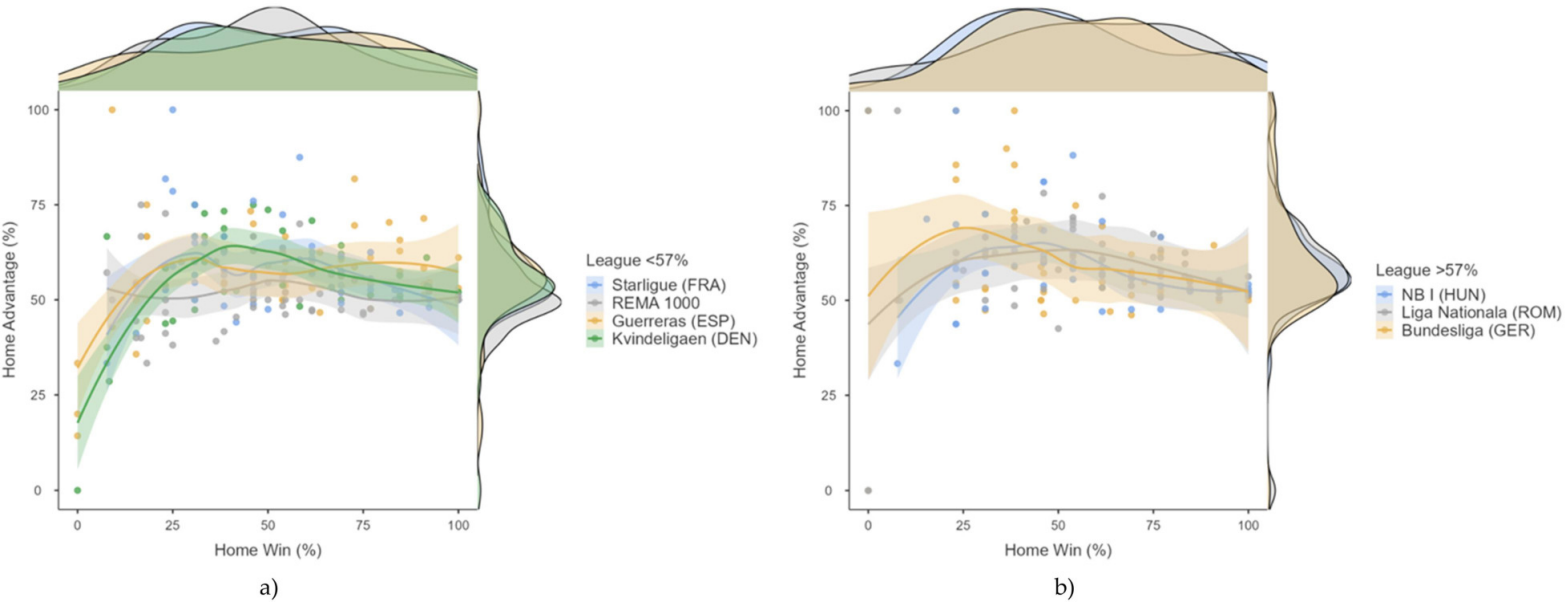
Graph showing HA percentages below and higher than 57. Each dot represents a team-season data point colored by league. The plot includes: 1) Scatter points for each observation; 2) Smoothed trend lines (with confidence intervals) for each league; (3) Marginal density plots showing the distributions of HW (top) and HA (right). **(a)** Below 57%; **(b)** higher than 57%.

Table 3 shows the descriptive data of HA and HW among the different teams in relation to league and team level. Figure 1 shows the relationship of HA and HW as a function of the league. Figure 2 shows a descriptive pattern of concomitant HW and HA. The Pearson correlation was not significant [*r*(380) = 0.020; *p* = 0.697], so any interpretation should be considered exploratory. The HA-HW relationship varies from league to league. Some leagues show high values between HW and HA [e.g., Guerreras Iberdrola (ESP), REMA 1000 (NOR)]. Others, such as NB I, show a non-linear trend, with a maximum of HA at moderate HW values and a slight decrease at very high winning percentages. In addition, some teams [especially in the Bundesliga (GER) and Kvindeligaen (DEN)] have relatively high HW but moderate or even low HA, suggesting a balanced home and away performance. HA distributions are tightly clustered between 45% and 75% for most leagues, although REMA 1000 (NOR) and Guerreras Iberdrola (ESP) show more dispersion. In the case of HW, REMA 1000 and NB I show higher densities around 60%–75% HW. Liga Națională (ROM) and Starligue (FRA) have flatter distributions, suggesting more balanced or unpredictable local win rates.

**Table 3:** Descriptive statistics for HA, and HW based on league and team level.

Home Advantage (%)																				
	HLT					MLT					LLT					Total				
	N	M	SD	IC—95%		N	M	SD	IC—95%		N	M	SD	IC—95%		N	M	SD	IC—95%	
				LL	UP				LL	UP				LL	UP				LL	UP
Bundesliga (GER)	11	51.19	3.80	43.71	58.67	16	57.02	3.15	50.82	63.22	27	66.67	2.43	61.89	71.44	54	58.29	1.84	54.68	61.90
Starligue (FRA)	9	50.25	4.21	41.98	58.52	20	55.73	2.82	50.18	61.28	26	62.24	2.47	57.38	67.11	55	56.07	1.88	52.38	59.77
Guerreras Iberdrola (ESP)	9	54.30	4.21	46.03	62.57	20	59.43	2.82	53.89	64.98	23	55.33	2.63	50.16	60.50	52	56.36	1.90	52.62	60.10
Kvindeligaen (DEN)	13	52.91	3.50	46.03	59.80	13	56.35	3.50	49.47	63.23	29	57.12	2.34	52.52	61.73	55	55.46	1.82	51.87	59.05
Liga Natională (ROM)	12	54.24	3.64	47.08	61.40	14	57.82	3.37	51.19	64.45	29	60.98	2.34	56.38	65.59	55	57.68	1.83	54.09	61.28
NB I (HUN)	14	51.98	3.37	45.35	58.61	9	56.28	4.21	48.01	64.55	33	63.53	2.20	59.21	67.85	56	57.26	1.94	53.45	61.08
REMA 1000 (NOR)	10	48.83	3.99	40.99	56.68	13	50.27	3.50	43.39	57.15	30	54.32	2.30	49.79	58.85	53	51.14	1.93	47.35	54.94
Total	78	51.96	1.45	49.11	54.80	105	56.13	1.27	53.63	58.63	197	60.03	0.90	58.25	61.81	380	57.42	13.13	52.63	63.67

Home win percentage (%)																				
	HLT					MLT					LLT					Total				
	N	M	SD	IC—95%		N	M	SD	IC—95%		N	M	SD	IC—95%		N	M	SD	IC—95%	
				LL	UP				LL	UP				LL	UP				LL	UP
Bundesliga (GER)	11	86.21	4.00	78.34	94.07	16	66.00	3.32	59.47	72.52	27	35.48	2.55	30.46	40.50	54	62.56	1.93	58.77	66.36
Starligue (FRA)	9	90.46	4.42	81.76	99.15	20	61.96	2.97	56.12	67.79	26	32.27	2.60	27.16	37.39	55	61.56	1.98	57.68	65.45
Guerreras Iberdrola (ESP)	9	89.28	4.42	80.58	97.97	20	68.46	2.97	62.63	74.30	23	25.63	2.77	20.19	31.07	52	61.12	2.00	57.19	65.06
Kvindeligaen (DEN)	13	90.98	3.68	83.74	98.21	13	63.26	3.68	56.03	70.50	29	31.90	2.46	27.05	36.74	55	62.05	1.92	58.27	65.82
Liga Natională (ROM)	12	88.89	3.83	81.36	96.42	14	69.69	3.55	62.72	76.66	29	34.37	2.46	29.53	39.22	55	64.32	1.92	60.53	68.10
NB I (HUN)	14	91.21	3.55	84.24	98.18	9	61.54	4.42	52.84	70.23	33	36.60	2.31	32.06	41.14	56	63.11	2.04	59.10	67.13
REMA 1000 (NOR)	10	83.33	4.19	75.08	91.58	13	57.29	3.68	50.05	64.52	30	33.76	2.42	29.00	38.52	53	58.13	2.03	54.14	62.11
Total	78	88.62	1.52	85.63	91.61	105	64.03	1.34	61.40	66.66	197	32.86	0.95	30.99	34.73	380	53.20	26.12	48.58	57.51

N, sample; M, mean; SD, standard deviation; HLT, high level teams; MLT, medium level teams; LLT, low level teams; IC95%, interval confidence 95%; LL, lower limit; UP, upper limit.

**Figure 3:**
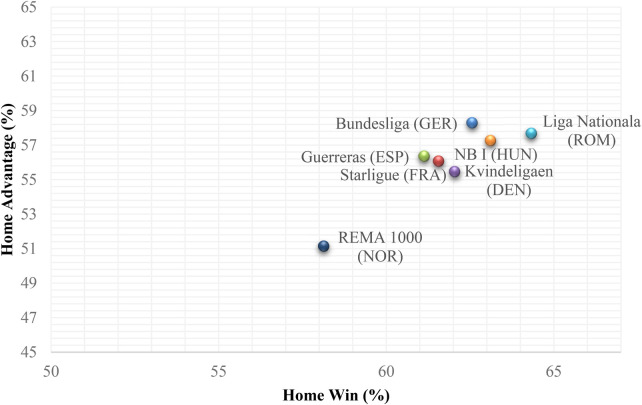
Scatter plot between HA and HW for each of the leagues.

The ANOVA test analysis revealed no differences between leagues in both HA (*F*_6,359_ = 1.55; *p* = 0.162; *η_p_*^2^ = 0.025; moderate) and HW (*F*_6,359_ = 0.95; *p* = 0.459; *η_p_*^2^ = 0.016; moderate) (see Figure 3).

**Figure 4:**
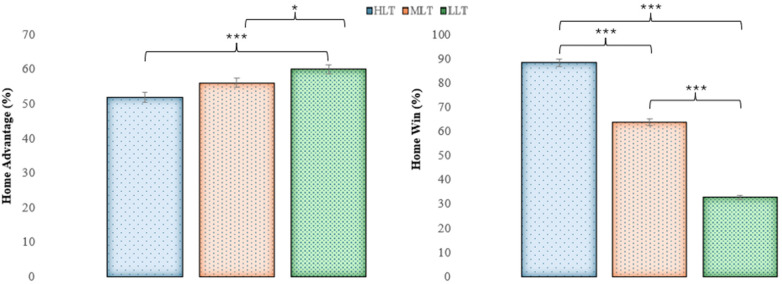
HA and HW according to team level. Description of significance values; * = *p* < 0.05; *** = *p* < 0.001; HLT, high level teams; MLT, medium level teams; LLT, low level teams.

Figure 4 shows the comparison of HA and HW according to team level. The ANOVA results showed significant differences for both HA (*F*_2,359_ = 11.79; *p* < 0.001; *η_p_*^2^ = 0.062; large) and HW (*F*_2,359_ = 532.35; *p* < 0.001; *η_p_*^2^= 0.748; large). *Post-hoc* multiple comparisons revealed significantly greater HA in LLT compared to MLT (+3.90%; *p* < 0.05; *d* *=* 0.36; small) and HLT (+8.07%; *p* < 0.001; *d* *=* 0.82; moderate). In relation to HW, HLT exhibited higher values than MLT (+24.59%) and LLT (+55.76%) (*p* < 0.001; *d* *=* 1.87; large, and *d* *=* 2.26; very large respectively). Furthermore, MLT showed greater HW than LLT at 31.17% (*p* < 0.001; *d* *=* 2.01; very large).

**Figure 5:**
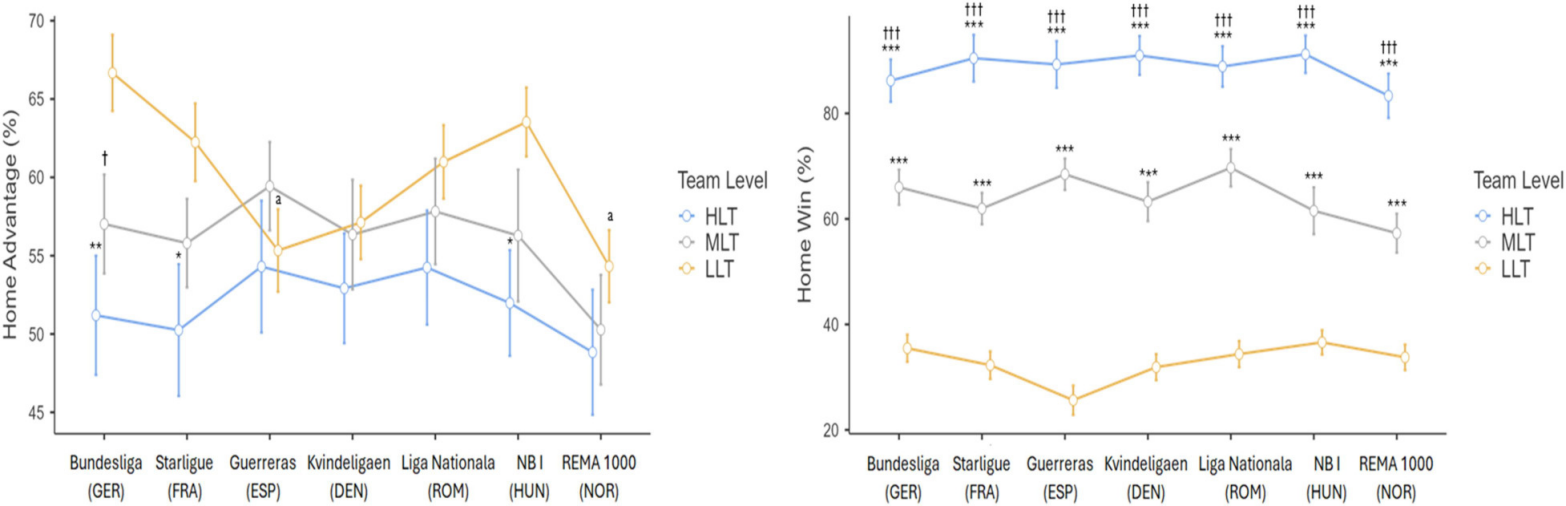
Differences in HA and HW in relation to team level between each league. Symbols to differentiate leagues: Bundesliga (GER). Symbols to differentiate between team levels: “*” indicates significant differences vs. LLT. “†” indicates significant differences vs. MLT. Significance level is indicated by the number of symbols: one symbol for *p* < 0.05, two for *p* < 0.01, and three for *p* < 0.001.

Regarding the interaction between league and the team level (Figure 5), there was no significant effect on HA (*F*_12,359_ = 0.98; *p* = 0.466; *η_p_*^2^ = 0.030; moderate) and HW (*F*_12,359_ = 1.44; *p* = 0.145; *η_p_*^2^ = 0.046; moderate). However, despite the overall model not yielding significant differences, the *post-hoc* tests did determine differences. In HA, the performance obtained with the LLT was significantly lower in the Guerreras Iberdrola (ESP) (dif. 11.33%; *p* < 0.05; *d* *=* 0.77; moderate), and REMA 1000 (NOR) (dif. 12.34%; *p* < 0.01; *d* *=* 0.82; moderate) than in the Bundesliga (GER). In HW-performance there were no significant differences. Furthermore, in HA comparing the internal team level of each league, the LLTs of Bundesliga (GER) (dif. 15.47%; *p* < 0.01; d = 1.12; moderate), Starligue (FRA) (dif. 11.99%; *p* < 0.05; d = 0.55; small), and NB I (HUN) (dif. 11.55%; *p* < 0.05; d = 0.54; small), had significantly higher values than the HLTs. Also, Bundesliga (GER) LLTs (dif. 9.64%; *p* < 0.05; d = 0.45; small) had significantly higher values than MLTs. In Guerreras Iberdrola (ESP), Kvindeligaen (DEN), Liga Natională (ROM) and REMA 1000 (NOR) there were no significant differences between their team levels (*p* > 0.05). In the variable HW, LLTs obtained significantly higher values than HLTs and MLTs (*p* < 0.001 in all conditions and leagues; d = 1.53–2.17; large and very large).

## 4 Discussion

The present study contributes to filling the gender and methodological gaps in HA research by providing cross-league comparison of elite women's handball. The design integrates seven of the most competitive European leagues and incorporates a hierarchical team-level classification, offering a multidimensional view of the phenomenon. This approach allows for a more realistic assessment of HA and HW beyond league boundaries and clarifies how competitive level interacts with contextual factors in high-performance women's sport. In doing so, the study expands the current understanding of performance asymmetries under comparable structural and cultural conditions, helping to bridge the persistent gender gap in HA research. Given the absence of direct team ability controls, the findings should be interpreted as descriptive patterns of home and away performance rather than isolated estimates of home advantage effects. This study provides the first comprehensive comparison of home advantage (HA) and home win percentage (HW) across seven elite European women's handball leagues, revealing that team competitive level (HLT > MLT > LLT) explains more variation than league context. The absence of significant inter-league differences, combined with consistent intra-league team hierarchies, advances existing research by demonstrating that structural quality differences dominate over national or cultural factors in modern women's handball.

The present research aimed: 1) to analyse the home advantage (HA) and home winning percentage (HW) in each of the main European women's handball leagues over the last four seasons; 2) to examine the impact of team level on HA and HW across these leagues during the seasons 2021–2025. The results showed that no significant differences were found in HA and HW between leagues. The absence of significant differences between leagues might indicate that the home advantage effect is a stable and universal feature of elite European women's handball, reflecting comparable competitive and contextual conditions between countries. However, significant differences by team level were observed: high level (HLT) and medium level (MLT) teams outperformed low level (LLT) teams in both metrics. The interaction between league and team level was limited, standing out only in HA for LLT teams, while in HW it was not significant. Within leagues, significant differences in HA were found in some leagues (Bundesliga (GER), Starligue (FRA), NB I (HUN), while in HW these differences were consistent between leagues and levels.

### 4.1 Why is there no difference between leagues?

The absence of significant differences in HA and HW between leagues might suggest a homogenisation of competitive environments in elite women's handball (18), already reported that HA in European women's leagues tends to be less pronounced than in men's leagues, which is consistent with other studies (33). Furthermore, Forrest et al. (34), suggested that HA differences between leagues are modulated by competitive balance, being higher in less balanced contexts.

The lack of significant differences in HA and HW between leagues in this research, although this was an exploratory analysis, could be explained by common structural factors in European women's competitions (35). Similarities in competition formats, the size of the facilities, and the post-COVID average attendance could have contributed to the observed similarity. Although the seasons analysed correspond to the post-pandemic period (2021–2022 to 2024–2025), the residual effects of COVID-19 may have contributed to the observed stability in HA and HW between leagues. During and immediately after the pandemic, several structural and behavioural changes occurred in professional handball: reduced attendance capacity, stricter travel logistics, and modified competition calendars. These factors could have attenuated traditional crowd-related advantages and promoted greater parity between home and away performances. The persistence of these trends in the post-COVID period suggests a possible structural normalisation of the competition environment rather than a temporary disruption (36–38). Furthermore, the increased professionalism and competitive balance in European women's handball, with a greater dispersion of talent between teams in different leagues, could have reduced the differences in HA/HW between countries (1, 39).

### 4.2 Differences in team level

Differences by team level highlight structural inequalities in competitiveness. LLT consistently showed higher HA and HW than HLT, which is consistent with previous findings in men's handball and other sports (22, 40, 41). This may be related to superior tactical cohesion, deeper rosters, and greater psychological resilience of HLTs when playing at home, as some research. In HA, the most marked contrasts between HLT and LLT, and between MLT and LLT, might indicate that weaker teams struggle more to take advantage of the benefits of the home court (42, 43). In LLT teams, lower HA values were observed together with poorer final standings, suggesting that home results and overall classification reflect a shared underlying performance pattern rather than a distinct effect of HA on league position. Combined with high HA and HW in HLT and MLT teams, this makes it difficult for LLT teams to score points in the final classification in away matches. The combination of these two variables may result in low positions in the final ranking. This finding is consistent with previous research indicating that HLT tend to maximise HA due to their tactical, physical and organisational superiority (44, 45). These teams tend to have better resources, such as access to specialised coaches, performance analysis technologies, and more advanced physical recovery programmes, allowing them to optimise their performance at home (46).

In addition, HLT tend to have more comfortable travel and more efficient logistics, reducing accumulated fatigue and improving their overall performance (47). In terms of HW, the differences between levels suggest that the intrinsic quality of the team, rather than the local context, is the main determinant of home wins. This might reinforce the idea that contextual conditions, such as crowd support or familiarity with the environment, amplify advantages when the team's capabilities are already superior (7, 48).

Our finding that HLT consistently outperform MLT and LLT in both HA and HW aligns with team heterogeneity models showing that higher-quality teams better exploit home contexts (12). However, without ability adjustment, we cannot determine whether: 1) HLT genuinely benefit more from home advantage, or 2) LLT appear disadvantaged primarily due to their baseline weakness. Football literature suggests team-specific HA varies systematically with quality: elite teams convert home contexts into points more efficiently through tactical execution and psychological resilience (49). Similar dynamics may operate in handball, where HLT's superior depth, coaching, and experience amplify location effects. Ability-adjusted analyses would be required to test this hypothesis formally.

### 4.3 Interaction between league and team level

Interaction effects between team level and league were minimal in HA and HW, which might reinforce the idea that team strength has a more dominant influence on performance outcomes than league-specific factors. However, in the LLT category, HA was significantly lower in Bundesliga (GER) compared to REMA 1000 (NOR) and Guerreras Iberdrola (ESP). This may reflect differences in structural parity: the German league shows greater disparity between top and bottom teams (e.g., budget gaps), potentially reducing competitiveness among LLTs even at home (21, 50).

National competitive structures seem to have converged, with the average level of international players becoming more homogeneous after COVID-19 (25, 51, 52). However, in Norway and Spain, this may reflect a greater competitive imbalance, with LLT relying more on the local environment to compete against superior opponents (53, 54).

### 4.4 Internal league differences according to team level

Intra-league analyses showed consistent and significant differences by team level. These findings underscore the internal stratification of leagues. Regarding HW, all leagues showed significant differences between HLT, MLT, and LLT, that might confirm the impact of team hierarchy on competitive success regardless of playing venue (55, 56).

Some of the leagues analysed show high attendance rates, which amplifies the home advantage effect (57, 58). Moreover, the differences were also clear, possibly associated with the budgetary disparity between clubs (59). In contrast, the differences in HW were the same in all leagues, which might reinforce the idea that team level is the main explanatory factor for home performance, beyond country or league structure.

Overall, the findings may suggest that while the women's elite handball landscape appears balanced across countries, internal disparities between team levels remain a key factor. As other authors have pointed out, structural causes such as economic disparities, squad depth, tactical maturity, and psychological preparedness may explain these results (60–62). Furthermore, the relatively reduced differences in HA and HW between leagues may reflect a trend toward greater equality and professionalism in women's sport, although internal league parity remains a challenge. Although differences between team levels and leagues were observed, these patterns should be interpreted cautiously. Without explicit control for team ability, it is not possible to determine whether these differences reflect contextual home advantage effects or underlying disparities in team strength. Therefore, the results should be understood as descriptive associations rather than causal relationships.

## 5 Limitations

This research has a number of limitations that should be considered when evaluating the findings. First, it was not feasible to precisely manage the impact of structural characteristics of sports facilities, such as the number of seating areas (whether one, two, three, or four) and the overall capacity of the arenas. Second, the amount of travel undertaken by the away team was not factored as a variable for analysis. Additionally, the connection between the population size of the team's home city and the home advantage was not specifically explored. It should be noted that playoff matches were excluded because the competition format and number of participating teams differ between leagues, which would compromise the comparability and homogeneity of the data. Furthermore, the playoff structure is not present in all leagues. However, the authors plan to specifically address HA and HW in playoff or decisive matches. Third, a methodological limitation of the present study is the partial endogeneity between the team-level classification (HLT, MLT, LLT) and the outcome variables (HA and HW). Teams were clustered according to their points obtained relative to the maximum possible points, a performance metric that is itself a function of the same match results used to compute HW and, indirectly, HA. Consequently, the observed differences in HA and HW across team levels cannot be interpreted as independent of overall competitive strength, but rather as patterns emerging from a joint structure in which performance, classification and home results are intrinsically linked. Future research should consider alternative approaches that explicitly model team ability (e.g., rating systems, team-level regression models) and derive HA estimates that are adjusted for underlying quality, thereby reducing this endogeneity. The observed patterns in HA and HW must be interpreted with consideration of their structural interdependence. Since HA is mathematically derived from the relative contribution of home points to total performance, and HW reflects the absolute frequency of home victories, systematic divergences between the two metrics may reflect construction effects rather than distinct underlying mechanisms. For instance, the more pronounced team-level differences in HW compared to HA could arise because HW directly measures win frequency (less sensitive to draws or narrow losses), while HA incorporates the full spectrum of match outcomes through points ratios. This interdependence suggests that our findings represent joint patterns of home and overall performance rather than independent effects of contextual factors. Future research should consider contextual factors such as the design of the arenas, actual attendance at games, the distance and time taken for visiting teams to travel, as well as the size of the local population. Also, future studies employing ability-adjusted models or alternative HA specifications (e.g., log5 or Pythagorean expectations) could further disentangle these relationships. This would enable a more comprehensive and accurate examination of home advantage and home win rates.

## 6 Practical applications

The practical applications derived from this study are relevant to coaches, analysts, managers, and decision makers of teams and federations in elite women's handball. Firstly, having objective information on the actual magnitude of the home advantage (HA) and the home win percentage (HW) allows for better adjustment of match preparation strategies, avoiding the overestimation of the local factor's impact, especially in lower-level teams or leagues with competitive balance. Technical staff can use these results to focus tactical and psychological work on variables that directly influence group performance, prioritising tactical adaptation and the management of competitive stress beyond the context of match location. Likewise, planning of travel, recovery, and logistics can benefit from the finding that the local environment no longer confers such a decisive advantage, allowing for the optimisation of resources and efforts. Perhaps the possibility of arriving at the match the day before and the possibility of training in the pavilion the day before the match for visiting teams (and the players' adaptation to the pavilion) could reduce the HA of the home teams. However, this measure will depend largely on the financial possibilities and the permission of both the competition and the home teams. For club and federation managers, the findings may guide policies aimed at promoting structural equity (budgets, youth development, facilities) and the professionalisation of technical staff, which are key to reducing inequalities and enhancing the overall competitiveness of the championship.

## 7 Conclusions

The results revealed that there are no significant differences between leagues in HA and HW, suggesting a certain competitive homogeneity between the main European women's competitions. However, team level was shown to be a key factor: low level teams (LLT) obtained higher HA but lower HW values than medium level (MLT) and high level (HLT) teams. Interaction between league and team level was limited, although the differences in HA between LLT teams of the Bundesliga (GER) and Guerreras Iberdrola (ESP) and REMA 1000 (NOR) were noticeable. In addition, intra-league analyses confirmed that differences by team level are consistent across most competitions, being more marked in HW.

These findings suggest that, although the structural conditions of women's leagues tend towards a certain inter-league equality, internal inequalities between teams continue to condition performance, especially about the benefits of home play. Future studies should delve deeper into the economic, tactical, and contextual factors that modulate these effects and explore whether these trends are sustained over time or are influenced by competitive rebalancing measures adopted by federations or leagues.

## Data Availability

The original contributions presented in the study are included in the article/Supplementary Material, further inquiries can be directed to the corresponding author.
