# Supplementary figures and images for: The women's side of home advantage: comparative analysis in the top seven handball leagues
Source: Front Sports Act Living. 2026 Apr 24;8:1798630. doi: 10.3389/fspor.2026.1798630 (PMC13155081; doi:10.3389/fspor.2026.1798630)

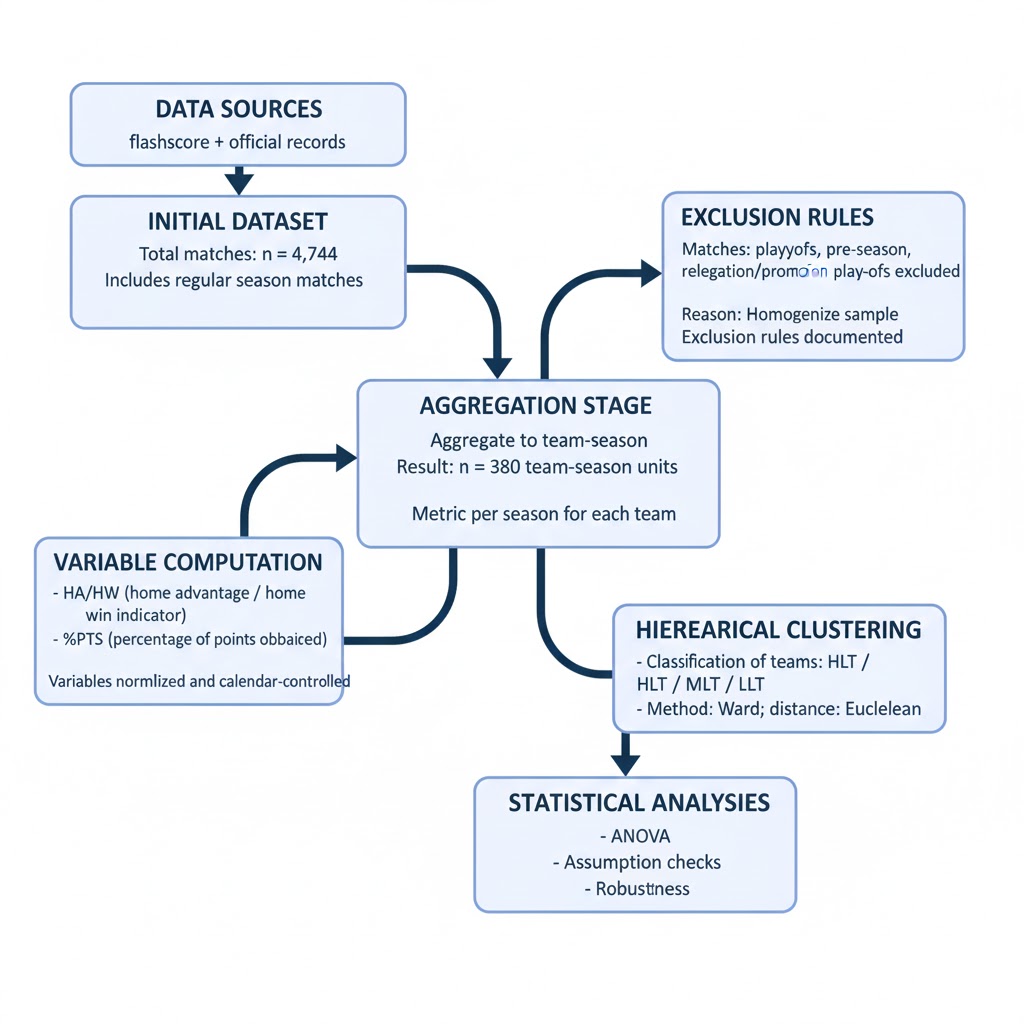

Supplement: Supplementary file 1 [file Datasheet1.zip › supplementary_materials/Flow Chart.jpg]
